# Knowledge and Risk Perception Regarding Skin Cancer in Immunosuppressed Patients: A Systematic Review and Meta-Analysis

**DOI:** 10.3390/ejihpe16080118

**Published:** 2026-08-17

**Authors:** Luisa Leonie Brokmeier, Sophia Haas, Laura Ilic, Wolfgang Uter, Markus Vincent Heppt, Olaf Gefeller, Isabelle Kaiser

**Affiliations:** 1 Department of Medical Informatics, Biometry and Epidemiology, Friedrich-Alexander-Universität Erlangen-Nürnberg, 91054 Erlangen, Germanyolaf.gefeller@fau.de (O.G.);; 2 Center for Health Services Research in Medicine, Department of Psychiatry and Psychotherapy, Uniklinikum Erlangen, Friedrich-Alexander-Universität Erlangen-Nürnberg, 91054 Erlangen, Germany; 3 Department of Dermatology, Uniklinikum Erlangen, Friedrich-Alexander-Universität Erlangen-Nürnberg, 91054 Erlangen, Germany; 4 Comprehensive Cancer Center Erlangen-European Metropolitan Area of Nürnberg (CCC ER-EMN), 91054 Erlangen, Germany; 5 Dermpath München, Laboratory for Dermatopathology, Oral Pathology and Molecular Pathology, 80335 Munich, Germany

**Keywords:** skin cancer, risk perception, knowledge, immunosuppression, systematic review, meta-analysis

## Abstract

Immunosuppressed patients have a substantially increased risk for developing skin cancer. Therefore, it is mandatory that such patients are adequately informed and aware of skin cancer and their increased risk to modify their behavior. Numerous studies have evaluated the topic, but no synthesis of their findings exists. The protocol of this systematic review was registered on PROSPERO (CRD42024618851) and its reporting followed the PRISMA-2020 guideline. To identify all studies assessing knowledge or perception of skin cancer in immunosuppressed patients, forward and backward citation tracking was employed in addition to an electronic literature search across five databases. For the risk of bias (ROB) assessment, the Joanna Briggs Institute checklist for prevalence studies was used. Quantitatively comparable outcomes were meta-analyzed, while all others were qualitatively synthesized. Thirty-two reports comprising 4214 patients were included. Acknowledging substantial heterogeneity between studies, just over two thirds of patients recalled having been informed about their increased skin cancer risk (pooled proportion: 68.34%, 95% confidence interval (CI): 55.48–78.90). Awareness of the increased risk for skin cancer varied widely (34.5–100%), and pooled mean Skin Cancer and Sun Knowledge scores were 14.77 (CI = 8.57–20.96). About half of those patients asked about risk factors for skin cancer knew that immunosuppression was among them. This indicates that immunosuppressed patients are insufficiently educated about their increased skin cancer risk. However, the high heterogeneity in the synthesized outcomes and the minimal proportion (3%) of low ROB ratings for the included studies call for further research to better understand the awareness of skin cancer risk in this vulnerable population.

## 1. Introduction

Some patients face lifelong dependence on immunosuppressive medications, e.g., to prevent organ rejection after transplantation or to suppress an overly active (auto-aggressive) immune system. However, this essential therapy increases the risk of various complications, including skin cancer. Studies have shown that organ transplant recipients (OTRs) are at significantly higher risk for skin malignancies, such as basal cell carcinoma, squamous cell carcinoma, and melanoma, compared to the general population (Rollan et al., 2022). For melanoma, it has been shown that chronic immunosuppression impedes the body’s capacity to identify and eradicate neoplastic melanocytes (Collins et al., 2019). A recent meta-analysis comprising data from 10 studies, including 22,415 heart transplant recipients, quantified a pooled relative risk of 2.21 (95% confidence interval (CI): 1.32–3.71) for heart transplant recipients to develop melanoma (Campillo et al., 2025). For both melanoma and keratinocyte carcinoma, comprising basal cell carcinoma and squamous cell carcinoma, ultraviolet (UV) radiation exposure is an important risk factor (Collins et al., 2019; Nanz et al., 2024). Since the incidence of skin cancer is high (Leiter et al., 2020) and immunosuppressed patients are at an even greater risk of developing skin cancer (Wheless et al., 2014), UV prevention among this vulnerable population seems imperative.

Understanding and increasing the awareness and risk perceptions of skin cancer is critical for developing effective education and intervention strategies aimed at early detection, prevention, and improved patient outcomes (Michie et al., 2011). Furthermore, both are essential parts of models of health behavior, such as the Behavior Change Wheel (Michie et al., 2011), the Health Belief Model (Becker, 1974), the Health Action Process Approach (Schwarzer, 2008), or the Transtheoretical Model (Prochaska & DiClemente, 1983). However, these models differ in their operationalization of the constructs of risk perception and awareness. The latter is often used interchangeably with knowledge, which in turn is being defined by awareness in several dictionaries (Trevethan, 2017). Trevethan (Trevethan, 2017) argues that both are on the same continuum of a knowledge domain, ranging from general awareness knowledge to detailed and specific knowledge. Indeed, the psychological constructs of awareness differ in the models mentioned, some referring to a general notion or even belief (Becker, 1974) and others to specific and consciously processed information (Prochaska & DiClemente, 1983). Similarly, risk perception is operationalized slightly differently: as the (subjectively) perceived vulnerability (Becker, 1974; Schwarzer, 2008) or as consciousness raising (Prochaska & DiClemente, 1983). Accordingly, worry is sometimes used in the measurement of risk perception instead (Sjöberg, 1998). In fact, it seems that they are different domains, with worry referring to an emotional state and risk perception to a cognitive process (Sjöberg, 1998). But again, the different models refer to different processes in this regard. In a change towards more preventative behavior, all these constructs are important. Educational programs, including those targeting individuals at risk, incorporate the dissemination of information about skin cancer risk as one component in order to improve sun protection behavior (Alonso-Belmonte et al., 2022; Wu et al., 2016). Thus, knowledge about skin cancer, including its risk factors, early symptoms, and preventive measures, may play a pivotal role in reducing the incidence of skin malignancies and improving survival rates among OTRs. Similarly, how OTRs perceive their own risk for skin cancer, and whether they take appropriate preventive actions, are key elements that influence the overall health outcomes. Despite this, the level of knowledge about skin cancer prevention and the perception of skin cancer risk among OTRs remain underexplored (Nagarajan et al., 2019).

This systematic review aims to synthesize existing research on the knowledge of skin cancer and the risk perceptions towards it among immunosuppressed patients. This will provide insights into the factors influencing the patients’ adherence to skin cancer prevention and guide the development of tailored educational programs and healthcare policies. The findings could also inform future research tackling the burden of skin cancer in this high-risk population.

## 2. Materials and Methods

This systematic review was part of a larger review project registered with PROSPERO (registration number: CRD42024618851). In this project, another review focusing on knowledge and risk perception regarding KC in lay people has already been published (Brokmeier et al., 2025), in which the general methods of this project have been described in detail. Therefore, this article will describe the procedure only briefly. In conducting and reporting this review, we followed PRISMA-2020 guideline (Moher et al., 2010) (see Supplementary Material Table S1).

### 2.1. Eligibility Criteria

Following the SPIDER search strategy (Cooke et al., 2012) studies were included as follows:

- Sample: Immunosuppressed individuals, e.g., transplant recipients.
- Phenomenon of Interest: knowledge about, risk perception, or attitudes towards skin cancer. This also includes awareness, familiarity, or beliefs, but not merely addressing sun-related knowledge or behavioral UV assessment.
- Design: Cross-sectional surveys, cohort studies, baseline data of intervention studies.
- Evaluation: Items had to be sufficiently described to ascertain they were distinctly assessing the phenomenon of interest.
- Research Type: Quantitative studies published in peer-reviewed journals.

Non-English or non-German studies were screened, assessed for eligibility, and extracted by reviewers with sufficient expertise in the respective language. All reviewers were native German speakers and combined had an adequate level of proficiency in English, French, Portuguese, Russian, and Serbian. Where necessary, translation tools were additionally used to clarify specific passages. Publications in other languages were excluded due to a lack of linguistic expertise among the reviewers. We further sorted out all studies that were published as conference abstracts, case reports, or dissertations. Publications on qualitative studies and reviews of previous studies were excluded, as well.

### 2.2. Search Strategy

We systematically searched the databases Medline (via PubMed), EMBASE (via Scopus), Web of Science, PsycArticles, and PsycINFO using a combination of keywords and MeSH terms regarding skin cancer, knowledge, and risk perception, as well as terms specifying the study design (details in the Supplementary Table S2). The literature search of the databases was not time-restricted and covered the entire period from the inception of each database until 9 September 2025.

We further used the method of citation tracking (Haddaway et al., 2022). Three systematic reviews (Fernandez-Ruiz et al., 2022; Nahar et al., 2020; Ziehfreund et al., 2019) and one study (Duarte et al., 2018) were used for backward citation tracking and the reference lists were extracted from Scopus. This database was also used for the forward citation tracking: All publications citing three relevant older studies (MacKie, 2004; Miles et al., 2005; Pfahlberg et al., 1997) on our research topic were extracted. Following the PRESS guideline (McGowan et al., 2016), members of the team reviewed the search protocol independent from its development.

Using an Endnote library, all references were allocated, and duplicates were eliminated. In the first screening phase, two researchers independently reviewed the titles and abstracts of this reference list for eligible articles, using Rayyan (Ouzzani et al., 2016). All articles considered eligible by at least one reviewer were included in the second, full text screening phase, maximizing sensitivity of the search process. Full texts were read independently by two researchers who decided about inclusion based on the predefined inclusion criteria. In case of disagreements, these were discussed until a consensus was reached, or a third researcher was consulted for a decision.

### 2.3. Data Extraction and Quality Assessment

Using the Systematic Review Data Repository Plus (SRDR+, https://srdrplus.ahrq.gov, accessed on 30 September 2025), two researchers independently extracted data on study design, setting, sample characteristics, item features, and results of the included studies. SRDR+ offers automatic detection of conflicts between two data extractions. Those were checked and consolidated. The data extraction template is provided in Supplementary Table S3.

To assess the risk of bias (ROB), the Joanna Briggs Institute (JBI) checklist for prevalence studies (Munn et al., 2015) was applied. After establishing decision criteria, studies were rated by two independent researchers. High concerns regarding items referring to selection bias, measurement bias, or an adequate sample size (set at *n* ≥ 100) led to an overall rating of high ROB. If items could not be rated due to missing information, ROB was classified as unclear. Conflicts were resolved by discussion until a consensus was reached.

### 2.4. Data Synthesis

Study characteristics and ROB ratings of the included studies were summarized in tables. Results were reported separately for the domains knowledge and attitude. Within these domains, items were thematically clustered to allow better comparison of the assessed outcomes. These subcategories were as follows: risk perception of skin cancer, awareness of an increased risk to develop skin cancer, concern about developing skin cancer, knowledge about skin cancer risk factors specific to immunosuppression, knowledge about general skin cancer risk factors, skin cancer knowledge scores, and other items. Due to high heterogeneity regarding outcome definition, wording of items, and scale assessment, the results were qualitatively synthesized, except for the following:

A meta-analysis was performed for the outcome “informed about skin cancer risk”, including all studies that assessed this outcome with comparable wording. One study (Ali & Cronin, 2025) reported separate proportions for two distinct patient populations and was therefore included twice. Furthermore, studies reporting skin cancer knowledge assessed on the same scale, namely the validated Skin Cancer and Sun Knowledge Score (SCSK) (Day et al., 2014) were synthesized in a meta-analysis pooling means. This questionnaire assesses general knowledge about skin cancer and sun protection and ranges from 0 to 25 points, with 25 equaling highest knowledge. Confidence intervals (CIs) were calculated using the Wilson method for the reported proportions of patients having been informed and with the Hartung and Knapp (Hartung & Knapp, 2001) method for means, respectively. Generalized linear mixed models (GLMM) with random effects were used to conduct the meta-analyses. The I^2^-statistic (range 0–100%, with high heterogeneity assumed for I^2^ ≥ 75%) (Higgins et al., 2003) was calculated to assess the between-study heterogeneity, as well as the between-study variance (τ^2^). To address a high heterogeneity, we calculated in addition to the conventional 95% CI the 95% prediction intervals (PI), which reflects the true range covering 95% of future study results for the outcome of interest (Guddat et al., 2012). The presence of potential publication bias or small-study effects was assessed by inspection of a funnel plot. In view of the limited number of studies using the SCSK score, publication bias was only assessed for the outcome “informed about skin cancer risk”. The meta-analyses were conducted in R version 4.2.2 (https://cran.r-project.org/) using the R packages ‘meta’ (Balduzzi et al., 2019) and ‘metafor’ (Viechtbauer, 2010).

## 3. Results

**Figure 1:**
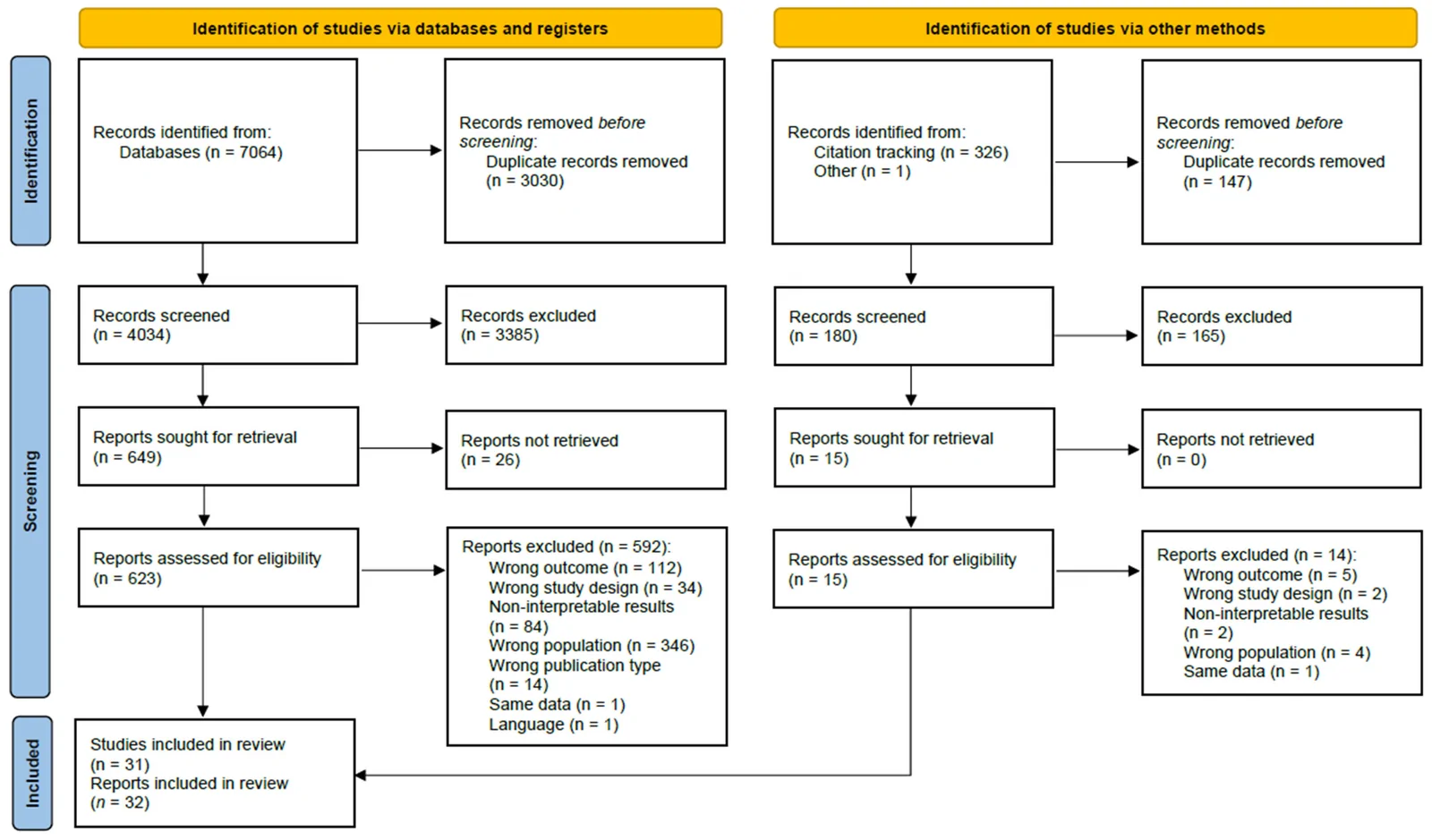
PRISMA flow-chart of the systematic literature review process.

The literature search yielded 7391 references. After removal of duplicates, 4214 studies were screened in the first phase and 638 in the second. Of those, 32 publications assessed immunosuppressed patients and were therefore included (for the PRISMA flow-chart see Figure 1). Two of these comprised the same sample (Robinson et al., 2015, 2016), resulting in 31 studies included.

Studies were published between 1999 and 2025. Most studies were conducted in the US (k = 13) and in Europe (k = 11), followed by Australia (k = 3), Turkey (k = 3), and Canada (k = 1). Overall, 4151 patients and 20 guardians of underage patients were assessed, with sample sizes between 20 (with 20 guardians) and 445 (Table A1). Age ranged from 6 to 94 years, 17.8–70% of the samples were female. All samples consisted of patients with iatrogenic immunosuppression. Most of the studies included patients with organ transplantation, only two assessed patients with inflammatory bowel disease and one comprised end-stage renal disease patients undergoing hemodialysis (Table A2). Eight studies provided details about the medication taken by the patients (Supplementary Table S4). A single study received a low ROB rating, while fifteen received the ratings unclear and high, respectively (Table A1).

### 3.1. Informed About Skin Cancer

**Figure 2:**
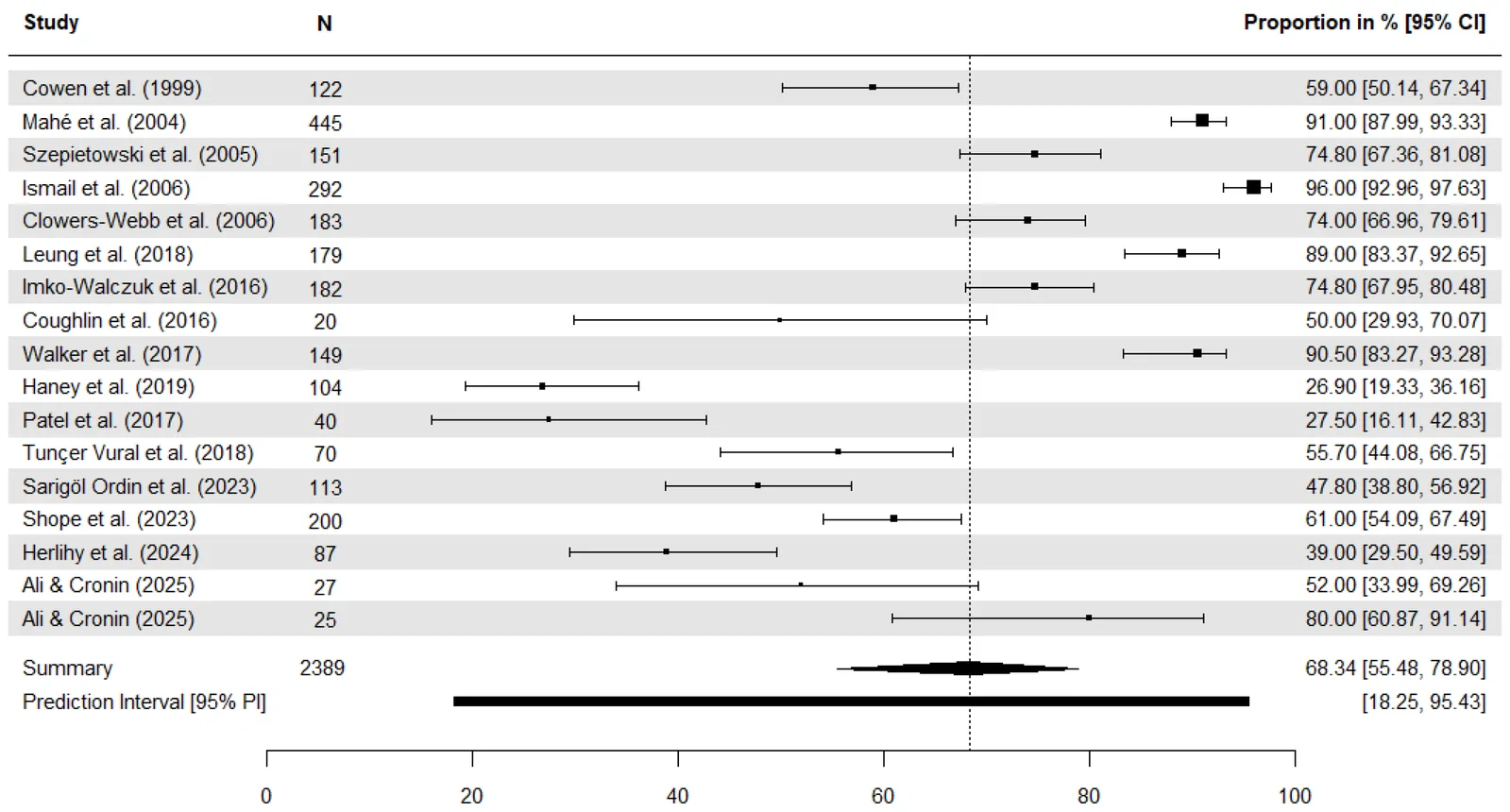
Forest plot with meta-analysis showing the proportions of having been informed about an increased skin cancer risk due to immunosuppression. The studies are chronologically ordered by assessment date. CI = Confidence interval.

Sixteen studies assessed whether patients had been informed about their increased skin cancer risk due to the immunosuppression and reported the responses in proportions (Supplementary Table S5). In four of those (Haney et al., 2019; Herlihy et al., 2024; Patel et al., 2017; Sarigöl Ordin et al., 2023), less than 50% of the sample indicated they had received such information (range: 27.5–47.8%). Two of these studies were conducted in Turkey (Haney et al., 2019; Sarigöl Ordin et al., 2023). On the other hand, in three studies (Ismail et al., 2006; Mahé et al., 2004; Walker et al., 2017), more than 90% of the patients reported being informed (range: 90.5–96%). A pooled proportion of 68.35% (CI = 55.48–78.90, PI = 18.25–95.43, τ^2^ = 1.26, I^2^ = 95.8%, *p* < 0.001, k = 17) of assessed patients reported having been informed about their increased risk for SC (Figure 2). The proportions of patients having been informed seemed to have decreased over time, since proportions were lower in more recent studies. The funnel plot (Supplementary Figure S1) shows a symmetrical distribution, thus providing no evidence for publication bias.

### 3.2. Risk Perception of Skin Cancer

The majority of patients (56.8–77.5%) agreed to the notion of being at risk for skin cancer (Parvathala et al., 2024; Wang et al., 2023) or recognized their personal skin cancer risk (median = 4 on a scale from 1 (strongly disagree) to 5 (strongly agree), (Robinson et al., 2014, Table 1). On the other hand, the latter item revealed rather low recognition of personal skin cancer risk in another sample (mean = 1 or 2, or median = 2 or 3 in Robinson et al. (2015, 2016)). When asked to rate the likeliness of getting skin cancer compared to an average OTR, answers were almost evenly distributed between agreement (38.2% and 35.6%), neutral (23.7% and 30.7%), and disagreement (38.1% and 33.7%) (Wang et al., 2023). When asked to compare their risk to others in general, most patients rated their skin cancer risk to be average (71%), and only 22% higher than average (Robinson & Rigel, 2004).

### 3.3. Awareness of an Increased Risk to Develop Skin Cancer

Whether patients were aware of their increased skin cancer risk was assessed in thirteen studies. Answers indicating awareness (Yes, [strongly] agree, or aware) ranged from 34.5% (Sarigöl Ordin et al., 2023) to 100% (O’Grady et al., 2020). “Don’t know” answers were options in three studies, chosen by 7% (Tavadia et al., 2006), 31.2% (Szepietowski et al., 2005), and 35.4% (Sarigöl Ordin et al., 2023) of respondents, respectively (see Table 2).

### 3.4. Concern About Developing Skin Cancer

Concern differed widely across the five studies that assessed it (Table 3): While 86% of patients reported to be concerned about developing skin cancer in one study (Tavadia et al., 2006), the median answers were 2 and 3 on a scale from 1 to 10 (low-high concern) in another (Robinson et al., 2016). In the remaining studies, patients expressed moderate concern (Patel et al., 2017; Robinson et al., 2014; Wang et al., 2023).

### 3.5. Knowledge About Skin Cancer Risk Factors Specific to Immunosuppression

Knowledge about the increased skin cancer risk due to immunosuppressive medication was assessed in three studies (Ismail et al., 2006; Thet et al., 2021; Wang et al., 2023), indicating that up to 40.7% of respondents (Wang et al., 2023) were unaware of this fact (Table 4). The one study that compared patients’ knowledge by age found older patients to be less informed about their increased risk (Thet et al., 2021). In two further studies, participants had to recognize immunosuppression as a risk factor for skin cancer in a list of possible items, which was achieved by 45.9–58% (Herlihy et al., 2024; Kimmel et al., 2016, Table 4). From a slightly different angle, patients were asked whether they knew their increased skin cancer risk was the main reason for additional photoprotection after a transplant, which 68% had understood (Ismail et al., 2006).

**Table 4:** Knowledge about skin cancer risk factors specific to immunosuppression. The publications are chronologically ordered by assessment date in the corresponding studies.

Publication	N	Items	Results	
Ismail et al. (2006)	292	Understanding that increased risk of skin cancer was the main reason extra photoprotective measures were important after a transplant	*n* = 199 (68%)	
Kimmel et al. (2016)	164	Which of the following factors increase melanoma risk?	Immunosuppression: *n* = 95 (58%) For the rest of the list of risk factors, see Table 5	
Thet et al. (2021)	KTR: 57 GD: 45		KTR	GD
		Do you know that immunosuppressive medications can increase the risk of developing skin cancers?	Yes: *n* = 56 (98.2%) No: *n* = 1 (1.8%)	Yes: *n* = 31 (70.5%) No: *n* = 13 (29.5%)
			Older participants were less likely aware of immunosuppressant-related skin cancer (OR = 0.92, 95%-CI = 0.87–0.97, *p* = 0.003).	
Wang et al. (2023)	POC: 118 Non-POC: 101		POC	Non-POC
		Is longer time on transplant anti-rejection meds a risk factor for skin cancer in OTRs?	Yes: *n* = 70 (59.3%) No: *n* = 48 (40.7%)	Yes: *n* = 60 (59.4%) No: *n* = 41 (40.6%)
		Is infection of the skin with HPV a risk factor for skin cancer in OTRs?	Yes: *n* = 45 (38.1%) No: *n* = 73 (61.9%)	Yes: *n* = 23 (22.8%) No: *n* = 78 (77.2%)
		Is being out in the sun a risk factor for skin cancer in OTRs?	Yes: *n* = 109 (92.4%) No: *n* = 9 (7.6%)	Yes: *n* = 98 (97%) No: *n* = 3 (3%)
		Is smoking tobacco a risk factor for skin cancer in OTRs?	Yes: *n* = 83 (70.3%) No: *n* = 35 (29.7%)	Yes: *n* = 51 (50.5%) No: *n* = 50 (49.5%)
		Is young age a risk factor for skin cancer in OTRs?	Yes: *n* = 41 (34.7%) No: *n* = 77 (65.3%)	Yes: *n* = 20 (19.8%) No: *n* = 81 (80.2%)
Herlihy et al. (2024)	87	Which of the following increase melanoma risk? (Agreement)	Immunosuppression: *n* = 40 (45.9%) For the rest of the list of risk factors, see Table 5	

OTR = Organ transplant recipients. KTR = Kidney transplant recipients. GD = Glomerular disease. HPV = Human papillomavirus. POC = People of Color.

**Table 5:** Knowledge about general skin cancer risk factors. The publications are chronologically ordered by assessment date in the corresponding studies.

Publication	N	Items	Results	
Tavadia et al. (2006)	118	People who work outdoors are at increased risk of developing skin cancer	Agree: 82% Don’t know: 8% Disagree: 8%	
		People who have had frequent foreign holidays are at increased risk of developing skin cancer	Agree: 74% Don’t know: 7% Disagree: 17%	
		People who burn easily in the sun have a reduced risk for developing skin cancer	Agree: 3% Don’t know: 14% Disagree: 81%	
Mahé et al. (2004)	445	Do you know why you need to protect your skin against the sun? If yes, why?	Yes: 75% of those: Risk of skin cancer: 47%	
Szepietowski et al. (2005)	151	Is there any relationship between sunlight exposure and skin cancers?	Yes: *n* = 61 (40.4%) No: *n* = 8 (5.3%) I don’t know: *n* = 82 (54.3%)	
Leung et al. (2018)	179	If I protect myself from the sun, I can avoid skin cancer	Agree: 74%	
Robinson et al. (2014)	103	How confident are you that sun protection can prevent the development of skin cancer? (scale: 1 not at all confident–5 extremely confident)	Median (25, 75%ile) [Range]: Intervention Group: 3 (2, 3) [1, 4] Standard Care: 3 (3, 4) [1, 4]	
Robinson et al. (2016)	170	Confidence in sun protection preventing skin cancer (scale: 1 not at all confident–5 extremely confident, 2 items)	Median (25, 75%ile) [Range]: Intervention Group: 4 (3, 6) [2, 10] Standard Care: 5 (3, 7) [2, 10]	
Walker et al. (2017)	149	Are you aware that sun exposure is the best-known cause of skin cancer?	Yes: *n* = 148 (99.3%)	
Haney et al. (2019)	104	Does sun exposure cause skin cancer?	Yes: *n* = 63 (60.6%) No: *n* = 9 (8.7%) I do not know: *n* = 32 (30.8%)	
Kimmel et al. (2016)	164	Which of the following factors increase melanoma risk?	None 0% (*n* = 0), Having lots of moles 46% (*n* = 76), Particular diets 10% (*n* = 17), Family history of skin cancer 77% (*n* = 126), Fair complexion 70% (*n* = 114), Alcohol use 9% (*n* = 14), Sunburns 80% (*n* = 131), Prolonged sun exposure 79% (*n* = 130), Smoking 30% (*n* = 50), Blue eyes 26% (*n* = 42), Green eyes 13% (*n* = 22), Red hair 26% (*n* = 42), Fair hair 29% (*n* = 47), Immunosuppression 58% (*n* = 95), All of the above: 24% (*n* = 40)	
		Skin cancer risk can be minimized by avoiding sun and using adequate sun protection	True: 97% False: 3%	
Ankudowicz et al. (2018)	105	Patients were asked to name at least 3 risk factors for cutaneous carcinogenesis	Able to name 3: *n* = 3 (2.9%) Able to name 2: *n* = 31 (29.5%) Able to name 1: *n* = 63 (60%) None: *n* = 8 (7.6%)	
Tunçer Vural et al. (2018)	70	Do you know hazardous consequences of sun exposure?	Yes, “relation with cancer”: *n* = 28 (40%) Yes, other: *n* = 10 (14.3%) No: *n* = 32 (45.7%)	
de Gálvez et al. (2019)	151	Using tanning beds before 30 years of age increases your risk of developing melanoma by 75%.	True: *n* = 126 (83%)	
		Ultraviolet radiation causes the skin to age more quickly and might result in the development of different types of skin cancers	True: *n* = 144 (95%)	
	152	Applying sunscreen is the best way to protect yourself from the sun and prevent skin cancer from developing.	False: *n* = 53 (35%)	
Thet et al. (2021)	KTR: 57; GD: 45	Do you think ultraviolet radiation can play a role in the occurrence of skin cancer?	Yes: KTR: *n* = 48 (84.2%); GD: *n* = 32 (76.2%) No: KTR: *n* = 9 (15.7%); GD: *n* = 10 (23.8%) Note: Participants with higher education were more likely aware of ultraviolet radiation-related skin cancer (OR = 1.50, 95%-CI = 1.15–1.95, *p* = 0.003)	
Wang et al. (2023)	POC: 118; Non-POC: 101		POC	Non-POC
		People who do not sunburn can get skin cancer	True: *n* = 81 (68.6%) False: *n* = 37 (31.4%)	True: *n* = 92 (91.1%) False: *n* = 9 (8.9%)
		People with darker skin can get skin cancer	True: *n* = 80 (67.8%) False: *n* = 38 (32.2%)	True: *n* = 98 (97%) False: *n* = 3 (3%)
Herlihy et al. (2024)	87	Protecting my skin from the sun will reduce my risk of skin cancer.	Agree: *n* = 85 (97.6%) Disagree: *n* = 0 (0%) Neutral: *n* = 2 (2.4%)	
		Which of the following increase melanoma risk? (Agreement)	Having lots of moles: *n* = 54 (64.3%); Family history of melanoma: *n* = 72 (82.7%); Fair complexion: *n* = 46 (52.8%); Blue eyes: *n* = 18 (20.7%); Green eyes: *n* = 16 (18.4%); Prolonged sun exposure: *n* = 72 (82.8%); Sunburns: *n* = 75 (86.2%); Blonde hair: *n* = 26 (29.9%); Red hair: *n* = 28 (32.1%); Inflammatory bowel disease: *n* = 53 (60.9%); Immunosuppression: *n* = 40 (45.9%)	
Parvathala et al. (2024)	39	Using sunscreen decreases your risk of skin cancer	Strongly agree: *n* = 19 (48.7%), Agree: *n* = 18 (46.2%), Uncertain: *n* = 2 (5.1%)	

KTR = Kidney transplant recipients. GD = Glomerular disease. POC = People of Color.

### 3.6. Knowledge About General Skin Caner Risk Factors

Patients were asked about their awareness of various risk factors for skin cancer (Table 5). In most studies (k = 7), patients were asked whether they knew that sunlight or UV radiation causes skin cancer (de Gálvez et al., 2019; Haney et al., 2019; Herlihy et al., 2024; Szepietowski et al., 2005; Thet et al., 2021; Tunçer Vural et al., 2018; Walker et al., 2017). In three of these, a rather low percentage of patients were aware of this relationship (40% (Tunçer Vural et al., 2018), 40.4% (Szepietowski et al., 2005), and 60.6% (Haney et al., 2019)), while in the remaining studies, percentages were higher (76.2% (Thet et al., 2021)-99.3% (Walker et al., 2017)). Aiming at the same topic, patients were asked whether sun protection could prevent skin cancer in further five studies (Kimmel et al., 2016; Leung et al., 2018; Mahé et al., 2004; Robinson et al., 2016; Robinson et al., 2014), with agreement ranging from 74% (Leung et al., 2018) to 97% (Kimmel et al., 2016). When explicitly asked whether sunscreen would decrease the risk for skin cancer, 94.9% agreed (Parvathala et al., 2024). In another study, only 35% were aware that sunscreens are not the best way to prevent skin cancer (de Gálvez et al., 2019). People of Color (POC) were less often aware of the fact that they could still develop skin cancer. While 97% of patients who were non-POC agreed to this, only 67.8% of POC patients did (Wang et al., 2023).

### 3.7. Skin Cancer Knowledge Scores

**Figure 3:**
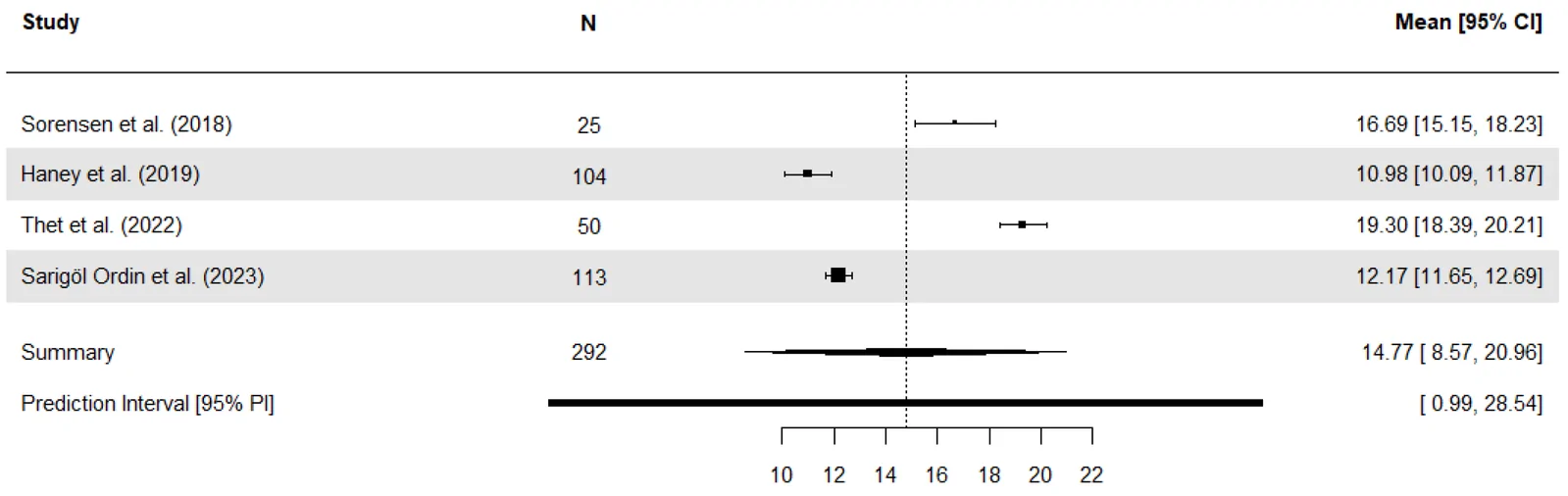
Forest plot with meta-analysis showing the pooled means of the Skin Cancer and Sun Knowledge scores. CI = Confidence interval.

Four studies (Haney et al., 2019; Sarigöl Ordin et al., 2023; Sorensen et al., 2018; Thet et al., 2022) used the SCSK score to assess knowledge about skin cancer. In the included studies, patients achieved mean scores on the SCSK of 10.98 (Haney et al., 2019) to 19.3 (Thet et al., 2022) points (Supplementary Table S6). A meta-analysis revealed a pooled mean of 14.77 (CI = 8.57–20.96, PI = 0.99–28.54, τ^2^ = 14.94, I^2^ = 98.7%, *p* < 0.001, k = 4; Figure 3). Two further studies developed their own score consisting of several skin cancer-related items, revealing rather high knowledge in one sample (mean proportion of correct answers: 91.7%) (Clowers-Webb et al., 2006), and low to medium knowledge in underage OTR and their guardians (Coughlin et al., 2017).

### 3.8. Other Items

Two items in the same study (Wang et al., 2023) assessed whether patients felt there was not much they could do to lower their chances of developing skin cancer (agreement: 8.9–18.7%) and whether they felt confident they would detect skin cancer (agreement: 75.4–81.2%, Supplementary Table S7). On the other hand, 39.6–50% of patients without a history of skin cancer knew what skin cancer would look like as opposed to 66.7–85.7% with a history of skin cancer (Farahbakhsh et al., 2024). Furthermore, 98.2% of the sample in Kimmel et al. (2016) were aware of the fact that skin cancer could not be healed without treatment, but only 1.2% knew that skin cancer could lead to death if untreated.

## 4. Discussion

This systematic search for studies on knowledge and risk perception regarding skin cancer with immunosuppressed patients yielded 32 eligible publications describing 31 different observational studies, comprising altogether 4151 patients. The samples consisted mainly of OTRs. The ROB assessment was concerning, attesting that only one study had a low risk of bias. The rest of the studies were split evenly between the ratings unclear and high. This stresses the need not only for more high-quality research but also for a more transparent and precise reporting of methods and results of a study. The assessment of outcomes in the individual studies was very heterogeneous, allowing mostly for a qualitative synthesis of the results only. One of the few outcomes assessed in a comparable manner across several studies was the proportion of patients who reported having been informed about their increased risk for skin cancer. Acknowledging the high structural heterogeneity between studies, we decided to quantitatively synthesize this outcome in a meta-analysis yielding a pooled estimate of just over two-thirds of patients who recalled having been informed.

Furthermore, there are geographical discrepancies in the proportion of patients being informed. Two of the four studies with proportions below 50 were conducted in Turkey (Haney et al., 2019; Sarigöl Ordin et al., 2023), and the third Turkish study reported only a slightly higher proportion of 55.7% (Tunçer Vural et al., 2018). One possible reason for a low proportion of patients being informed about their risk might be a lack in education accompanying the immunosuppression therapy. A recent systematic review (Chang et al., 2024) covering population studies assessing the role of health literacy in skin cancer found that high levels of health literacy are associated with better preventive behavior. In general, health literacy in Turkey seems to be rather low (Özkan et al., 2020), supporting our interpretation. However, patients’ recall problems might be another reason, as suggested by Imko-Walczuk et al. (2016). In addition to geographical heterogeneity, temporal differences are also apparent. Any temporal developments should, however, be cautiously interpreted due the substantial heterogeneity in study populations in different regions and healthcare systems. With a few exceptions (Walker et al. (2017) and the HIV-negative subsample of Ali and Cronin (2025)), the proportions of patients being informed in studies conducted after 2014 are lower than in older studies. One possible reason is the increasing time pressure faced by treating physicians due to economic constraints. Separating such a possibly existing temporal effect from a geographical effect is not possible due to their potential overlap.

Proportions of patients that were aware of their increased risk of developing skin cancer were even lower in most studies (Ali & Cronin, 2025; Cowen & Billingsley, 1999; Imko-Walczuk et al., 2016; Sarigöl Ordin et al., 2023; Szepietowski et al., 2005) than the proportions of patients in these studies having been informed about their increased risk. Knowledge about immunosuppression being a risk factor for skin cancer was rather low (around 50%), stressing the need for more education in this regard. Thus, some authors (e.g., Cowen & Billingsley, 1999; Patel et al., 2017) argue that patients must be repeatedly informed about their increased skin cancer risk due to immunosuppression. This might be especially important for older patients, who seem to be less informed about SC risk factors (Ankudowicz et al., 2018) and, more specifically, about immunosuppression being a risk factor for skin cancer (Thet et al., 2021). The fact that incidence increases with age (Niino & Matsuda, 2021) underlines the importance of adequately informing older patients. Another vulnerable group prone to insufficient information seem to be People of Color (POC): They were more likely to feel protected from skin cancer by their darker skin and failed more skin cancer knowledge items (Wang et al., 2023). Even though overall incidence of skin cancer is lower in POC compared to individuals with fair complexion, their malignancies are often more advanced at diagnosis (Zakhem et al., 2022). This highlights the need to increase awareness in this patient group.

Overall knowledge about skin cancer was measured in four studies (Haney et al., 2019; Sarigöl Ordin et al., 2023; Sorensen et al., 2018; Thet et al., 2022) with the validated and therefore comparable SCSK scale. Patients in these studies achieved a pooled mean score of 14.77 points. The scale itself ranges from 0 to 25 points, and the SCSK validation sample reached means of 18.35 (women) and 18.06 (men) (Day et al., 2014). Thus, we can infer that the pooled mean determined in this study is rather low. It would be desirable that patients on immunosuppressive medication, who are at increased risk for skin cancer, should achieve higher knowledge scores than the general population. However, the opposite seems to be the case. This is in line with the lower-than-expected rate of informed patients and stresses further the need for better education.

It might be most effective to transfer information about the increased risk by a specialist, i.e., the dermatologist. Some studies found better adherence to sun-protective behavior strategies in patients who had visited a dermatological clinic or had been advised by a dermatologist compared to those not informed by a specialist. This should be considered in future interventions. Furthermore, previous studies have underlined the importance of well-trained specialists’ communication towards their patients to increase adherence (Haskard Zolnierek & DiMatteo, 2009). Regular interdisciplinary liaison between mainly nephrologists and dermatologists appears mandatory. It might not be sufficient to simply convey information, but it seems to be important by whom and how (Haskard Zolnierek & DiMatteo, 2009). Nevertheless, mixed results regarding the efficacy of health education and resulting preventive behavior have been found: Some studies discuss that knowledge and sun protection did not correlate (e.g., Imko-Walczuk et al., 2016), and that “hassle” and a lack of time could be barriers (Farahbakhsh et al., 2024). Apart from the studies covered by our systematic review there is a substantial amount of literature (e.g., Allen & Damian, 2022; Reyes-Marcelino et al., 2021; Usher-Smith et al., 2018) on how knowledge and risk-feedback translate into sun protection and skin cancer prevention, including systematic reviews of educational and tailored interventions and several randomized trials. Overall, these studies show only modest effects of increasing knowledge on intentions and short-term preventive behaviors. Thus, it should not be expected that increasing adherence to sun protection can simply be accomplished by informing about skin cancer risks. Achieving behavioral changes needs a more complex multi-component strategy in which knowledge transfer is only one element. This finding, repeatedly made in studies of children, adolescents, and the general population, is also valid for vulnerable subpopulations of patients under immunosuppressive medication covered by this systematic review.

This review faces some limitations that mandate caution in interpreting the results. We cannot rule out some publication bias, which should lead to fewer studies with small sample sizes. However, the studies included in this review comprised both small and larger samples, indicating that this should not have led to a bias in this case. The funnel plot for the outcome “informed about skin cancer” also showed no signs of publication bias. In addition, our literature search may have overlooked published studies on the topic. We have minimized this limitation by searching in five different literature databases using a sensitive search string and by implementing additionally efficient backward and forward citation tracking techniques. Furthermore, the high heterogeneity of the extracted outcomes contributed to broad prediction intervals, reflecting the uncertainty of the pooled proportions and means. These pooled estimates should therefore be interpreted cautiously as they reflect (weighted) averages over highly diverse studies that may have addressed slightly different underlying concepts. Most outcomes were assessed with items that were worded or scaled so differently that a quantitative synthesis was impossible. However, our qualitative approach does provide a structured overview of the current evidence published to highlight educational gaps in immunosuppressed patients regarding skin cancer.

While this review identified important gaps in knowledge and risk perception regarding skin cancer among immunosuppressed patients, it was not designed to evaluate specific interventions or to scrutinize structural deficits in health systems. Questions related to how these findings can be translated into actionable strategies and implementation frameworks to fill these gaps remain important areas for future research.

## 5. Conclusions

To our knowledge, this is the first systematic review with meta-analysis summarizing studies on skin cancer risk perception and knowledge in immunosuppressed patients, a population particularly vulnerable to developing skin cancer. Acknowledging substantial heterogeneity between studies, almost a third of questioned patients included in this review did not confirm to having been informed about their increased skin cancer risk. Consequently, there were mixed results regarding their risk perception, indicating that this was not particularly high, and immunosuppression was only known as a risk factor for skin cancer by about 50% of patients. Furthermore, general skin cancer knowledge seemed to be lower than average. Our results call for improved patient communication and education to increase risk perception and knowledge about skin cancer. It should be better verified that immunosuppressed patients have understood their increased risk and that they know about risk factors and protective measurements.

## Figures and Tables

**Table 1:** Risk perception of skin cancer. The publications are chronologically ordered by assessment date in the corresponding studies.

Publication	N	Items	Results	
Robinson and Rigel (2004)	200	Do you think your risk of skin cancer or melanoma is (a) Higher than the average (b) About average (c) Less than average (d) Don’t know	Higher than average: 22% About average: 71% Less than average: 7%	
Robinson et al. (2014)	103	I am at risk of developing skin cancer. (scale: 1 strongly disagree–5 strongly agree)	Median (25, 75%ile) [Range]: Intervention Group: 4 (3, 4) [1, 4] Standard Care: 4 (2, 5) [1, 4]	
Robinson et al. (2015)	170	I am at risk of developing skin cancer. (scale: 1 strongly disagree–5 strongly agree)	Non-Hispanic white (*n* = 62): m = 2 (SD = 1.0) Hispanic/Latino (*n* = 48): m = 1 (SD = 0.6) Non-Hispanic black (*n* = 60): m = 1 (SD = 0.7)	
Robinson et al. (2016)	170	I am at risk of developing skin cancer. (scale: 1 strongly disagree–5 strongly agree)	Median (25, 75%ile) [Range]: Intervention Group: 2 (1, 3) [1, 5] Standard Care: 3 (2, 5) [1, 5]	
Wang et al. (2023)	POC: 118; Non-POC: 101		POC	Non-POC
		I am at risk for skin cancer	Strongly agree: 35 (29.7%) Somewhat agree: 32 (27.1%) Neutral: 14 (11.9%) Somewhat disagree: 14 (11.9%) Strongly disagree: 23 (19.5%)	Strongly agree: 47 (46.5%) Somewhat agree: 23 (22.8%) Neutral: 15 (14.9%) Somewhat disagree: 9 (8.9%) Strongly disagree: 7 (6.9%)
		Compared to the average OTR, I am more likely to get skin cancer	Strongly agree: 18 (15.3%) Somewhat agree: 27 (22.9%) Neutral: 28 (23.7%) Somewhat disagree: 21 (17.8%) Strongly disagree: 24 (20.3%)	Strongly agree: 20 (19.8%) Somewhat agree: 16 (15.8%) Neutral: 31 (30.7%) Somewhat disagree: 15 (14.9%) Strongly disagree: 19 (18.8%)
Parvathala et al. (2024)	40	I am at risk of skin cancer.	Strongly agree: 18 (45.0%), Agree: 13 (32.5%), Uncertain: 8 (20.0%), Disagree: 0 (0.0%), Strongly disagree: 1 (2.5%)	

m = Mean. SD = Standard deviation. POC = People of Color. OTR = Organ transplant recipients.

**Table 2:** Awareness of an increased risk to develop skin cancer. The publications are chronologically ordered by assessment date in the corresponding studies.

Publication	N	Items	Results
Cowen and Billingsley (1999)	122	Did you know that immunosuppressants increase the risk of skin cancer?	Yes: *n* = 61 (50%) No: *n* = 61 (50%)
Tavadia et al. (2006)	118	If you have a heart transplant then you have a higher risk of developing skin cancer	Agree: 89% Don’t know: 7% Disagree: 2%
Szepietowski et al. (2005)	151	Are renal transplant recipients especially predisposed for skin cancer development?	Yes: *n* = 103 (68.2%) No: *n* = 1 (0.6%) I don’t know: *n* = 47 (31.2%)
Imko-Walczuk et al. (2016)	182	How aware are the patients about their higher risk of skin cancer?	Aware: *n* = 94 (51.6%)
Sachse et al. (2016)	26	Being aware of the increased risk of developing skin cancer	Aware: *n* = 21 (81%)
Haney et al. (2019)	104	Do organ transplants increase the risk of skin cancer?	Yes: *n* = 84 (80.8%) No: *n* = 20 (19.2%)
Kimmel et al. (2016)	164	Are you at increased risk of having skin cancer because of your IBD and the medications that you take for the treatment of IBD?	Strongly agree: *n* = 39 (23.6%) Agree: *n* = 62 (37.6%) Neutral: *n* = 56 (33.9%) Disagree: *n* = 1 (0.6%) Strongly disagree: *n* = 6 (3.6%)
Patel et al. (2017)	40	Before transplant, were you aware of higher risk of skin cancer post-transplant?	Yes: *n* = 23 (57.5%) No: *n* = 17 (42.5%)
O’Grady et al. (2020)	87	Knowledge of skin cancer risks	All patients included were aware of their increased risk of skin cancer (100%)
Sarigöl Ordin et al. (2023)	113	Does transplantation increase the risk of skin cancer?	Yes: *n* = 39 (34.5%) No: *n* = 34 (30.1%) No idea: *n* = 40 (35.4%)
Shope et al. (2023)	200	Patients’ recognition of their increased skin cancer risk	Yes: *n* = 146 (73%) No: *n* = 54 (27%) Subgroup BMTR: Although most allograft recipients (75.3%) were aware that they were at increased risk for skin cancers, most autograft recipients (55.6%) were not aware.
Herlihy et al. (2024)	87	Are you aware of an increased risk of skin cancer in IBD?	Yes: *n* = 46 (52.4%) No: *n* = 30 (34.5%) Unsure: *n* = 11 (13.1%)
		Are you aware of an increased risk of skin cancer with some IBD treatments?	Yes: *n* = 50 (57.5%) No: *n* = 28 (32.2%) Unsure: *n* = 9 (10.3%)
Ali and Cronin (2025)	52 (HIV-positive: 27, HIV-negative: 25)	I am at an increased risk of skin cancer.	Yes: HIV-positive: *n* = 13 (48%), HIV-negative: *n* = 16 (64%)

IBD = Inflammatory bowel disease. BMTR = Bone marrow transplant recipients.

**Table 3:** Concern about developing skin cancer. The publications are chronologically ordered by assessment date in the corresponding studies.

Publication	N	Items	Results	
Tavadia et al. (2006)	118	Considering the problems I have had with my health in the past, I am not too concerned about developing skin cancer	Agree: 8% Don’t know: 6% Disagree: 86%	
Robinson et al. (2014)	103	How concerned are you about developing a skin cancer at some point in your life? (scale: 1 no concern–5 extremely concerned)	Median (25, 75%ile) [Range]: Intervention Group: 3 (2, 3) [1, 5] Standard Care: 3 (2, 4) [1, 5]	
Robinson et al. (2016)	170	Concern about developing skin cancer (scale: 1 no concern–5 extremely concerned, 2 items)	Median (25, 75%ile) [Range]: Intervention Group: 2 (1, 3) [1, 10] Standard Care: 3 (2, 5) [1, 10]	
Patel et al. (2017)	19; 4 patients erroneously omitted question	If aware of higher risk, how concerned?	Very concerned: *n* = 0 (0%) Moderately concerned: *n* = 8 (42.1%) Somewhat concerned: *n* = 3 (7.5%) A little bit concerned: *n* = 6 (15%) Not concerned: *n* = 2 (5%)	
Wang et al. (2023)	POC: 118; Non-POC: 101		POC	Non-POC
		I worry about getting skin cancer	Strongly agree: *n* = 31 (26.3%) Somewhat agree: *n* = 19 (16.1%) Neutral: *n* = 7 (5.9%) Somewhat disagree: *n* = 19 (16.1%) Strongly disagree: *n* = 42 (35.6%)	Strongly agree: *n* = 15 (14.9%) Somewhat agree: *n* = 28 (27.7%) Neutral: *n* = 14 (13.9%) Somewhat disagree: *n* = 23 (22.8%) Strongly disagree: *n* = 21 (20.8%)

POC = People of Color.

## Data Availability

The original contributions presented in this study are included in the article/Supplementary Materials. Further inquiries can be directed to the corresponding author.

## Supplementary Materials

The following supporting information can be downloaded at: https://www.mdpi.com/article/10.3390/ejihpe16080118/s1, Table S1. PRISMA 2020 Checklist; Table S2. Search string by Database; Table S3. Data extraction template (SRDR+); Table S4. Reported medication for immunosuppression; Table S5. Proportions of patients having been informed about their elevated skin cancer risk due to the immunosuppression; Figure S1: Funnel plot for the outcome “informed about skin cancer risk” (*n* = 17*); Table S6. Skin cancer knowledge scores; Table S7. Other items.

## Appendix A

### Appendix A.1

**Table A1:** Study details and results of risk of bias (ROB) assessment for all 31 studies ordered chronologically by assessment date of the corresponding studies.

Publication	Sample Size (% [Number] of Females)	Method of Recruitment and Information Assessment	Assessment Date	Country	ROB
Cowen and Billingsley (1999)	122 (40% [*n* = 49] ^a^)	Participants were recruited during follow-up visits to a renal transplant clinic administered oral questionnaire	n.r. *	USA	unclear
Tavadia et al. (2006)	118 (17.8% [*n* = 21])	Participants were recruited during follow-up visits to a cardiac transplant clinic self-administered questionnaire	July–April 2001 **	Scotland (UK)	unclear
Mahé et al. (2004)	445 (Sex ratio (men/women): 1.6)	Recruitment of consecutive patients at a renal transplant clinic self-administered questionnaire	March–May 2003 *	France	unclear
Robinson and Rigel (2004)	200 (50% [*n* = 100] ^a^)	Participants were recruited from a transplant registry (telephone contact) telephone survey	May 2003 *	USA	unclear
Szepietowski et al. (2005)	151 (39.7% [*n* = 60])	Participants were recruited during routine visits to a renal transplant clinic self-administered questionnaire	n.r. *	Poland	unclear
Ismail et al. (2006)	292 (43% [*n* = 125])	Participants were recruited from a renal transplant department database (postal contact) self-administered questionnaire	August 2004–April 2005 *	England	low
Clowers-Webb et al. (2006)	202 (41.1% [*n* = 83] ^a^)	Participants were recruited during visits to a transplant clinic for dermatologic consultation self-administered questionnaire	2006 **	USA	unclear
Leung et al. (2018)	179 (45.25% [*n* = 81])	Participants were recruited from hospitals’ transplant registries (postal contact) telephone interview	December 2008–February 2009 *	Australia	high
Imko-Walczuk et al. (2016)	182 (45.6% [*n* = 83])	Participants were recruited from a transplant center patient list self-administered questionnaire	December 2008–June 2011 *	Poland	unclear
Sachse et al. (2016)	26 (27% [*n* = 7])	Participants were recruited among attendees of a transplant summer camp for transplant recipients standardized questionnaires and telephone interviews	2011 **	Austria and Germany	high
Robinson et al. (2014)	103 (34.0% [*n* = 35])	Participants were recruited from a hospital database of transplant patients receiving ambulatory care self-administered questionnaire	May–July 2013 **	USA	high
Coughlin et al. (2017)	20 pairs of pediatric OTRs (55% [*n* = 11]) and guardians (70% [*n* = 14])	Participants were recruited during routine follow-up visits to a transplant clinic or during hospital admissions self-administered questionnaire	June 2013–January 2014 **	USA	high
Robinson et al. (2015, 2016)	170 (40.6% [*n* = 69] ^a^)	Participants were recruited from a transplant patient list (telephone contact before scheduled appointment) self-administered questionnaire (via tablets)	May–July 2014 **	USA	high
Walker et al. (2017)	149	Partcipants were recruited from a transplant patient list (postal contact) self-administered questionnaire	n.r. *	Canada	high
Haney et al. (2019)	104 (29.8% [*n* = 31])	Participants were recruited during routine follow-up visit to a liver transplant clinic self-administered questionnaire	March and September 2016 *	Turkey	unclear
Sorensen et al. (2018)	25 (24% [*n* = 6])	Participants were recruited during routine visits to a cardiac transplant clinic self-administered questionnaire	October–November 2016 **	USA	high
Kimmel et al. (2016)	164 (63% [*n* = 103] ^a^)	Participants were recruited from a gastroenterology clinic patient list (via email, online sources, and referral by healthcare providers) self-administered questionnaire	2016 *	USA	unclear
Patel et al. (2017)	40	Participants were recruited at a transplant center self-administered questionnaire	2016 **	USA	high
Ankudowicz et al. (2018)	105 (45.7% [*n* = 48] ^a^)	Participants were recruited from the patient lists of two nephrologic centers self-administered questionnaire	n.r. *	Poland	unclear
Tunçer Vural et al. (2018)	70 (21.4% [*n* = 15])	Recruitment of consecutive patients during routine follow-up visits at a transplant clinic self-administered questionnaire	January–March 2017 **	Turkey	high
de Gálvez et al. (2019)	166 (28.9% [*n* = 48])	Participants were recruited among participants of the World Transplant Games 2017 self-administered questionnaire	2017 *	Spain (Athletes worldwide)	unclear
O’Grady et al. (2020)	87	Participants were recruited from a transplant patient list self-administered questionnaire	n.r. *	Ireland	high
Thet et al. (2021)	112	Participants were recruited from the patient list of a nephrology center self-administered questionnaire	April–December 2019 *	Australia	unclear
Sarigöl Ordin et al. (2023)	113 (40.7% [*n* = 46] ^a^)	Participants were recruited at two outpatient clinics self-administered questionnaire	June 2019–February 2020 *	Turkey	unclear
Thet et al. (2022)	50 (42.0% [*n* = 21])	Recruitment of consecutive patients attending a nephrology center self-administered questionnaire	November 2020–April 2021 **	Australia	high
Shope et al. (2023)	200	Participants were recruited during routine follow-up visits self-administered questionnaire	April–November 2021 *	USA	unclear
Wang et al. (2023)	219 (44.8% [*n* = 98] ^a^)	Participants were recruited from a transplant center patient list (telephone contact of a random sample) computer-assisted telephone interviews	n.r. *	USA	high
Farahbakhsh et al. (2024)	107 (42.1% [*n* = 45])	Participants were recruited during a consultation visit at a dermatology clinic self-administered questionnaire	n.r. *	USA	unclear
Herlihy et al. (2024)	87 (52% [*n* = 45])	Participants were recruited during ambulatory care visits to a gastroenterology clinic self-administered questionnaire	n.r. *	Ireland	high
Parvathala et al. (2024)	91 (39.3% [*n* = 35])	Participants were recruited during visits to a dermatology clinic self-administered questionnaire	n.r. **	USA	high
Ali and Cronin (2025)	52 (36% [*n* = 19])	Participants were recruited from a renal transplant center database self-administered questionnaire	April 2023 *	UK	high

If no assessment date was provided, it was dated back 2 years from the publication date. ROB = Risk of Bias. n.r. = not reported. ^a^ calculated from information provided in the study. * Cross-Sectional; ** Baseline in Intervention Studies.

### Appendix A.2

**Table A2:** Composition of study samples with regard to type of recruited patients, distribution of age and skin type and time since transplantation for all 31 studies ordered chronologically by assessment date of the corresponding studies.

Publication	Patient Type *n* (%)	Age in Years Mean (m) ± SD	Skin Type ^a^ *n* (%)	Time Since Transplantation Mean (m) ± SD
Cowen and Billingsley (1999)	Organ transplantation (kidney)	m = 50 (range: 25–80)	Caucasian: 110, African American: 7, Hispanic: 3, Asian: 1, Middle Eastern descent: 1	m = 3.1 years (range: 1 week–27 years).
Tavadia et al. (2006)	Organ transplantation (cardiac)	m = 53.5 (range: 17–73)	n.r.	m = 4.7 years (range: 0.08–15)
Mahé et al. (2004)	Organ transplantation (kidney)	m = 48.2 ± 12.3	II: 13.3%; III–IV: 75.3%; V–VI: 11.5%	m = 12.5 ± 8.9 years
Robinson and Rigel (2004)	Organ transplantation ^b^	m = 47 (range: 26–67)	I–II: 8%; III or IV: 78%; V or VI: 14%	m = 2.9 years.
Szepietowski et al. (2005)	Organ transplantation (kidney)	m = 43.7 ± 11.4 (range: 19–69)	n.r.	m = 3.2 ± 3.6 years (range: 1 month to 20 years) (35 (23.2%) patients had had renal transplantation within 2 years before the survey, 60 (39.7%) patients had had transplantation in the period from 2 to 5 years before the examination and 56 (37.1%) patients were investigated 5 and more years after transplantation.
Ismail et al. (2006)	Organ transplantation (kidney)	m = 52	I: 22; II: 56; III: 124; IV: 86 missing: 4	n.r.
Clowers-Webb et al. (2006)	Organ transplantation (liver: 109 (54.0%), kidney: 51 (25.2%), heart: 44 (21.8%), pancreas: 3 (1.5%), lung: 3 (1.5%), heart & lung: 1 (0.5%), other: 1 (0.5%); some patients had more than one transplant)	m = 54.3 ± 14.6 (range: 11–76)	I: 24; II: 72; III: 70 IV: 29; V: 4; VI: 1 Unknown: 1	m = 6.3 ^c^
Leung et al. (2018)	Organ transplantation (kidney)	m = 54 ± 11 (range: 21–76)	Highly sensitive (burn only): 24%; Moderately sensitive (burn, then tan): 47%; Not sensitive (tan only): 29%	m = 10.6 ± 8.2 years
Imko-Walczuk et al. (2016)	Organ transplantation (kidney)	m = 49.7 (range: 21 to 78)	n.r.	m = 6.3 years
Sachse et al. (2016)	Organ transplantation (kidney: 17 (65.4%), liver: 6 (23.1%), heart: 1 (3.8%), kidney & liver: 2 (7.7%))	m = 16.1 (range: 13–22)	n.r.	n.r.
Robinson et al. (2014)	Organ transplantation (kidney)	m = 54 (range: 44–62)	White: 46 (44.7%); Latino/African American: 57 (55.3%)	n.r.
Coughlin et al. (2017)	Organ transplantation (kidney: 11 (55%), liver: 7 (35%), lung: 2 (10%), heart: 1 (5%); 1 patient had both liver and kidney transplants)	pOTR: m = 13 (range: 10–17); Guardians: m = 42 (range: 26–59)	pOTR: I: 2 (11%); II: 4 (21%); III: 4 (21%), IV: 4 (21%), V: 4 (21%), VI: 1 (5%)	m = 7 years (range: 2–13 years)
Robinson et al. (2015, 2016)	Organ transplantation (kidney)	m = 50.0 ± 13.4 ^c^	Non-Hispanic white: 62; Hispanic/Latino: 48; Non-Hispanic black: 60	m = 17.7 ± 15.2 months ^c^ (range: 2–24 months)
Walker et al. (2017)	Organ transplantation ^b^	n.r.	n.r.	n.r.
Haney et al. (2019)	Organ transplantation (liver)	m = 53.2 ± 11.8	I–II: 15 (14.4%); III–IV: 89 (85.6%)	m = 7.82 ± 4.39 years
Sorensen et al. (2018)	Organ transplantation (heart)	18 (72%) between the ages of 51 and 70	n.r.	range: less than 6 months to nearly 20 years
Kimmel et al. (2016)	Inflammatory bowel disease (Crohn’s disease: 109 (67%), ulcerative colitis: 51 (31%), indeterminate colitis: 4 (2%))	m = 43.5	I–II: 153 (94%); III–IV: 10 (5%)	n.r.
Patel et al. (2017)	Organ transplantation (kidney: 35 (87.5%), liver: 4 (10%), intestine: 1 (2.5%); 1 patient had both liver and kidney transplants)	n.r.	n.r.	up to 2 months
Ankudowicz et al. (2018)	End-stage renal disease undergoing hemodialysis	m = 60.8 (range: 25–94)	I: 11 (10.5%); II: 58 (55.2%); III–IV: 36 (34.3%)	Dialysis Time: <5 years: *n* = 7 (66.7%); 5–10 years: *n* = 21 (20%); >10 years: *n* = 14 (13.3%)
Tunçer Vural et al. (2018)	Organ transplantation (kidney: 55 (78.6%), liver: 15 (21.4%))	m = 36.3 ± 13.3 (range: 18–63)	II: 22 (31.4%); III: 38 (54.3%); IV: 10 (14.3%)	m = 6.4 ± 5.5 years (range: 1–23 years)
de Gálvez et al. (2019)	Bone marrow and organ transplantation (kidney: 79 (47.6%), lung: 12 (7.2%), heart: 31 (18.7%), liver: 33 (19.9%), bone marrow: 13 (7.8%), pancreas: 1 (0.6%))	m = 48 (range: 6–78)	I: 5 (3.1%); II: 71 (43.6%); III: 63 (38.7%); IV: 23 (14.1%); V: 1 (0.6%)	n.r.
O’Grady et al. (2020)	Organ transplantation (kidney)	m = 56 ± 12.51	I–II: The majority of patients IV: 2	m = 13.73 ± 15 years
Thet et al. (2021)	Organ transplantation (kidney; 61 (54.5%)) and GD (51 (45.5%))	m ± SD (range): KTR: 56.0 ± 14.3 (25–86); GD: 58.2 ± 17.3 (20–89)	KTR: I: 9 (14.8%); II: 12 (19.7%); III: 18 (29.5%); IV: 13 (21.3%); V: 1 (1.6%); VI: 1 (1.6%) GD: I: 4 (6.2%); II: 7 (10.8%); III: 17 (26.2%); IV: 13 (20.0%); V: 1 (1.5%); VI: 2 (3.1%)	n.r.
Sarigöl Ordin et al. (2023)	Organ transplantation (kidney: 82 (72.6%), liver: 31 (27.4%))	m = 46.63 ± 13.24	I: 8 (7.1%); II: 21 (18.6%); III: 17 (15.0%); IV: 60 (53.1%); V: 7 (6.2%)	m = 65.16 ± 64.69 months (range: 1–270 months)
Thet et al. (2022)	Organ transplantation (kidney; 25 (50%)) and GD (25 (50%))	Median age: 62, SD = 13.5 (range: 20–78)	Caucasian: 47 (94.0%); Asian: 3 (6.0%)	Median = 62.0 months (interquartile range: 36.8–108.5)
Shope et al. (2023)	Bone marrow and organ transplantation (bone marrow: 100 (50%), kidney: 59 (29.5%), liver: 16 (8%), heart: 18 (9%), lung: 7 (3.5%))	<16 years: *n* = 30 (15%) 16–25 years: *n* = 22 (11%) 26–49 years: *n* = 43 (21.5%) 50–65 years: *n* = 53 (26.5%) >65 years: *n* = 52 (26%)	Caucasian:122 (61%); African American: 59 (29.5%)	<6 months: *n* = 82 (41%) 6 months–1 year: *n* = 35 (17.5%) 1–5 years: *n* = 51 (25.5%) 5–10 years: *n* = 15 (7.5%) 10+ years: *n* = 17 (8.5%)
Wang et al. (2023)	Organ transplantation (POC: heart: 29 (24.6%), kidney: 27 (22.9%), liver: 39 (33.1%), lung: 23 (19.5%); Non-POC: heart: 20 (19.8%), kidney: 28 (27.7%), liver: 32 (31.7%), lung: 21 (20.8%))	POC: m = 54.0 ± 12.9; Non-POC: m = 56.8 ± 13.1	POC: I: 4 (3.4%); II: 2 (1.7%); III: 12 (10.2%); IV: 25 (24.8%); V: 3 (3.0%); VI: 16 (13.6%) Non-POC: I: 5 (5.0%); II: 26 (25.7%); III: 42 (41.6%); IV: 34 (28.8%); V: 50 (42.4%); VI: 0	POC: Mean = 2.6 ± 1.7 years Non-POC: Mean = 2.5 ± 1.5 years
Farahbakhsh et al. (2024)	Organ transplantation (kidney: 60 (56.1%), lung: 20 (18.7%), liver: 12 (11.2%), heart: 6 (5.6%), kidney & pancreas: 3(2.8%), kidney & liver: 3 (2.8%), kidney & lung: 1 (0.9%), double lung: 1 (0.9%), heart & lung: 1 (0.9%))	m = 56 (range: 21–85)	n.r.	<5 year: *n* = 21 (19.6%), 5–10 years: *n* = 39 (36.4%), 11–15 years: *n* = 26 (24.3%), 16–20 years: *n* = 10 (9.3%), >21 years: *n* = 11 (10.3%)
Herlihy et al. (2024)	Inflammatory bowel disease (Crohn’s disease: 56 (62.8%), ulcerative colitis: 30 (34.9%), indeterminate colitis: 1 (2.3%))	16–24: 12.9% (*n* = 11) 25–34: 27.1% (*n* = 23) 35–54: 37.2% (*n* = 32) 55–74: 18.8% (*n* = 16) ≥75: 3.5% (*n* = 3)	All Caucasian with the majority having fair or very fair skin (*n* = 67, 77%)	n.r.
Parvathala et al. (2024)	Organ transplantation (heart: 14 (15.2%), liver: 42 (45.7%), lung: 12 (13.0%), kidney: 28 (30.4%), pancreas: 2 (2.2%))	At least 18 years old (no further information provided)	American Indian/Alaskan Native: 3 (5.1%), Asian: 15 (25.4%), Black/African American: 2 (3.54%), White: 33 (55.9%), Other: 6 (10.2%)	n.r.
Ali and Cronin (2025)	Organ transplantation (Kidney; 27 (52%) HIV-positive)	median (HIV-positive): 55 (range: 43–74) median (HIV-negative): 54 (range: 44–72)	HIV-positive: Asian: 1 (4%); Black: 19 (70%); White: 7 (26%) HIV-negative: Asian: 1 (4%); Black: 17 (68%); White: 7 (28%)	HIV-positive: m = 7.4 ± 6.0 years; Median = 4.6 years (range: 0.7–23.6 years) HIV-negative: m = 7.7 ± 6.3 years; Median= 5.5 years (range: 0.7–23.7 years)

^a^ Roman numbers refer to Fitzpatrick Skin Types. ^b^ No information about transplant type reported in the study. ^c^ calculated from information provided in the study. n.r. = not reported. SD = standard deviation. pOTR = Pediatric organ transplant recipients. KTR = Kidney transplant recipients. GD = Glomerular disease. POC = People of Color.
