# Supplementary material for: Knowledge and Risk Perception Regarding Skin Cancer in Immunosuppressed Patients: A Systematic Review and Meta-Analysis
Source: Eur J Investig Health Psychol Educ. 2026 Aug 17;16(8):118. doi: 10.3390/ejihpe16080118 (PMC13512661; doi:10.3390/ejihpe16080118)
Supplement: Supplementary file 1 [file ejihpe-16-00118-s001.zip › ejihpe-4372948-supplementary.pdf]

Supplementary

Table S1: PRISMA 2020 Checklist

Section and Topic	Item #	Checklist item	Location where item is reported
**TITLE**			
Title	1	Identify the report as a systematic review.	Title
**ABSTRACT**			
Abstract	2	See the PRISMA 2020 for Abstracts checklist.	Abstract
**INTRODUCTION**			
Rationale	3	Describe the rationale for the review in the context of existing knowledge.	p. 2-3 (Introduction)
Objectives	4	Provide an explicit statement of the objective(s) or question(s) the review addresses.	p. 2-3 (Introduction)
**METHODS**			
Eligibility criteria	5	Specify the inclusion and exclusion criteria for the review and how studies were grouped for the syntheses.	p. 3 (section 2.1)
Information sources	6	Specify all databases, registers, websites, organisations, reference lists and other sources searched or consulted to identify studies. Specify the date when each source was last searched or consulted.	p. 3-4 (section 2.2)
Search strategy	7	Present the full search strategies for all databases, registers and websites, including any filters and limits used.	Supplement Table S2
Selection process	8	Specify the methods used to decide whether a study met the inclusion criteria of the review, including how many reviewers screened each record and each report retrieved, whether they worked independently, and if applicable, details of automation tools used in the process.	p. 3-4 (section 2.2)
Data collection process	9	Specify the methods used to collect data from reports, including how many reviewers collected data from each report, whether they worked independently, any processes for obtaining or confirming data from study investigators, and if applicable, details of automation tools used in the process.	p. 4 (section 2.3)
Data items	10a	List and define all outcomes for which data were sought. Specify whether all results that were compatible with each outcome domain in each study were sought (e.g. for all measures, time points, analyses), and if not, the methods used to decide which results to collect.	p. 4 (section 2.4)
	10b	List and define all other variables for which data were sought (e.g. participant and intervention characteristics, funding sources). Describe any assumptions made about any missing or unclear information.	p. 4 (section 2.3)
Study risk of bias assessment	11	Specify the methods used to assess risk of bias in the included studies, including details of the tool(s) used, how many reviewers assessed each study and whether they worked independently, and if applicable, details of automation tools used in the process.	p. 4 (section 2.3)
Effect measures	12	Specify for each outcome the effect measure(s) (e.g. risk ratio, mean difference) used in the synthesis or presentation of results.	p. 4 (section 2.4)

Section and Topic	Item #	Checklist item	Location where item is reported
Synthesis methods	13a	Describe the processes used to decide which studies were eligible for each synthesis (e.g. tabulating the study intervention characteristics and comparing against the planned groups for each synthesis (item #5)).	p. 4 (section 2.4)
	13b	Describe any methods required to prepare the data for presentation or synthesis, such as handling of missing summary statistics, or data conversions.	p. 4 (section 2.4)
	13c	Describe any methods used to tabulate or visually display results of individual studies and syntheses.	p. 4 (section 2.4)
	13d	Describe any methods used to synthesize results and provide a rationale for the choice(s). If meta-analysis was performed, describe the model(s), method(s) to identify the presence and extent of statistical heterogeneity, and software package(s) used.	p. 4 (section 2.4)
	13e	Describe any methods used to explore possible causes of heterogeneity among study results (e.g. subgroup analysis, meta-regression).	NA
	13f	Describe any sensitivity analyses conducted to assess robustness of the synthesized results.	NA
Reporting bias assessment	14	Describe any methods used to assess risk of bias due to missing results in a synthesis (arising from reporting biases).	p. 4 (section 2.4)
Certainty assessment	15	Describe any methods used to assess certainty (or confidence) in the body of evidence for an outcome.	p. 4 (section 2.4)
**RESULTS**			
Study selection	16a	Describe the results of the search and selection process, from the number of records identified in the search to the number of studies included in the review, ideally using a flow diagram.	p. 5 (Results); Figure 1
	16b	Cite studies that might appear to meet the inclusion criteria, but which were excluded, and explain why they were excluded.	NA
Study characteristics	17	Cite each included study and present its characteristics.	Table A1
Risk of bias in studies	18	Present assessments of risk of bias for each included study.	Table A1
Results of individual studies	19	For all outcomes, present, for each study: (a) summary statistics for each group (where appropriate) and (b) an effect estimate and its precision (e.g. confidence/credible interval), ideally using structured tables or plots.	p. 5-13 (Results)
Results of syntheses	20a	For each synthesis, briefly summarise the characteristics and risk of bias among contributing studies.	p. 5-13 (Results)
	20b	Present results of all statistical syntheses conducted. If meta-analysis was done, present for each the summary estimate and its precision (e.g. confidence/credible interval) and measures of statistical heterogeneity. If comparing groups, describe the direction of the effect.	p. 5-13 (Results)
	20c	Present results of all investigations of possible causes of heterogeneity among study results.	NA
	20d	Present results of all sensitivity analyses conducted to assess the robustness of the synthesized results.	NA
Reporting biases	21	Present assessments of risk of bias due to missing results (arising from reporting biases) for each synthesis assessed.	p. 5-13

Section and Topic	Item #	Checklist item	Location where item is reported
			(Results)
Certainty of evidence	22	Present assessments of certainty (or confidence) in the body of evidence for each outcome assessed.	p. 5-13 (Results)
**DISCUSSION**			
Discussion	23a	Provide a general interpretation of the results in the context of other evidence.	p. 14-15 (Discussion)
	23b	Discuss any limitations of the evidence included in the review.	p. 14-15 (Discussion)
	23c	Discuss any limitations of the review processes used.	p. 15-16 (Discussion)
	23d	Discuss implications of the results for practice, policy, and future research.	p. 15-16 (Discussion)
**OTHER INFORMATION**			
Registration and protocol	24a	Provide registration information for the review, including register name and registration number, or state that the review was not registered.	p. 3 (Methods)
	24b	Indicate where the review protocol can be accessed, or state that a protocol was not prepared.	p. 3 (Methods)
	24c	Describe and explain any amendments to information provided at registration or in the protocol.	NA
Support	25	Describe sources of financial or non-financial support for the review, and the role of the funders or sponsors in the review.	p. 16
Competing interests	26	Declare any competing interests of review authors.	p. 17
Availability of data, code and other materials	27	Report which of the following are publicly available and where they can be found: template data collection forms; data extracted from included studies; data used for all analyses; analytic code; any other materials used in the review.	p. 16

Table S2. Search string by database

**Pubmed**
(Knowledge [tiab] OR Attitude* [tiab] OR “Health Practice” [tiab] OR “Health Education” [tiab] OR “Attitude Health” [tiab: ~2] OR Awareness [tiab] OR Optimism [tiab] OR “risk perception” [tiab: ~2] OR “Risk Severity” [tiab]) AND (“skin neoplasms” [tiab] OR keratosis [tiab] OR “basal cell carcinoma” [tiab] OR “squamous cell carcinoma” [tiab] OR melanoma [tiab] OR “skin cancer” [tiab: ~2] OR nonmelanoma [tiab] OR “non-melanoma” [tiab] OR “cutaneous malignancies” [tiab] OR “malignant skin tumor” OR “epidermal cancer” [tiab] OR “keratinocyte carcinoma” [tiab]) AND (interview* [tiab] OR survey* [tiab] OR cross-sectional [tiab] OR questionnaire* [tiab])
**Web of Science**
(TS=(knowledge) OR TS=("health literacy") OR TS=(“health education”) OR TS=(attitude* NEAR/2 health) OR TS=(awareness) OR TS=(optimism) OR TS=(risk NEAR/2 perception*) OR TS=(“risk severity”)) AND (TS=(“skin neoplasms”) OR TS=(keratosis) OR TS=(“basal cell carcinoma”) OR TS=(“squamous cell carcinoma”) OR TS=(melanoma) OR TS=(“skin cancer”) OR TS=(cancer NEAR/2 skin) OR TS=(nonmelanoma) OR TS=(“non-melanoma”) OR TS=(“cutaneous malignancies”) OR TS=(“malignant skin tumor”) OR TS=(“epidermal cancer”) OR TS=(“keratinocyte carcinoma”)) AND (TS=interview* OR TS=survey OR TS=cross-sectional OR TS=question*)
**Scopus**
(TITLE-ABS-KEY (knowledge OR "health literacy" OR “health education” OR (attitude* w/2 health) OR awareness OR optimism OR (risk w/2 perception*) OR “risk severity”) AND TITLE-ABS-KEY ("skin neoplasms" OR keratosis OR "basal cell carcinoma" OR "squamous cell carcinoma" OR melanoma OR (cancer w/2 skin) OR nonmelanoma OR "non-melanoma" OR "cutaneous malignancies" OR "malignant skin tumor" OR "epidermal cancer" OR "keratinocyte carcinoma") AND TITLE-ABS-KEY (survey* OR cross-sectional OR interview* OR questionnaire*)) AND (EXCLUDE (DOCTYPE , "re"))
**CINAHL/PsycInfo/PsycArticles (via Ebsco Host)**
AB (knowledge OR health literacy OR health education OR attitude* health OR awareness OR optimism OR risk perception* OR risk severity) AND AB (skin neoplasms OR keratosis OR basal cell carcinoma OR squamous cell carcinoma OR melanoma OR skin cancer OR nonmelanoma OR non-melanoma OR cutaneous malignancies OR malignant skin tumor OR epidermal cancer OR keratinocyte carcinoma) AND AB (survey* OR cross-sectional OR interview* OR questionnaire*)

Table S4. Reported medication for immunosuppression. The publications are chronologically ordered by assessment date in the corresponding studies.

Publication	Immuno-suppressive medication
Cowen and Billingsley (1999)	Many patients were taking a combination of immunosuppressants, most commonly cyclosporine and prednisone, but several had taken azathioprine in the past, and several others were taking FK506 and mycophenolate.
Clowers-Webb et al. (2006)	Prednisone: 104, Cyclosporine: 92, Tacrolimus: 81, Azathioprinesodium: 61, Mycophenolatemofetil: 38, Sirolimus: 5, Muronomab-CD3(OKT3): 2, Antilymphocyteglobulin: 1, Other: 13<br>Number of drugs being taken: 1: 66, 2: 76, 3: 58, 4: 2
Coughlin et al. (2017)	Mean number of current immunosuppressant medications, (SD): 2 (0.65)
Haney et al. (2019)	40 (38.5%) were taking tacrolimus as immunosuppressive therapy
Kimmel et al. (2016)	current or past treatment<br>- with immunomodulators (which include Imuran/Azathioprine and 6-mercaptopurine): 105<br>- with biologics: 103
Thet et al. (2021)	61 KTR and 22 GD patients were maintained on immunosuppressive agents<br>Tacrolimus+mycophenolate sodium/mycophenolate mofetil+prednisolone: 40; Cyclosporin+mycophenolate sodium/mycophenolate mofetil+prednisolone: 4; Tacrolimus+azathioprine+prednisolone: 3; Sirolimus+mycophenolate sodium/mycophenolate mofetil+prednisolone: 3; Tacrolimus+prednisolone: 3; Cyclosporin+prednisolone: 3; Mycophenolate sodium/mycophenolatemofetil +prednisolone: 8; Azathioprine+prednisolone: 4; Mycophenolate sodium/mycophenolate mofetil: 6; Cyclosporin: 2; Azathioprine: 1; Others: 5
Thet et al. (2022)	Median time since the first dose of immunosuppressants: 62 months (interquartile range, IQR = 36.8 108.5 months)
Herlihy et al. (2024)	On active treatment for their IBD: 85 (96.5%).<br>Approximately one-third of patients were currently on an immunomodulator (azathioprine or 5-mercaptopurine) and a further 29% of patients had been previously. Approximately two thirds of patients were being treated with a biologic (65.8%). A total of 17 (19.5%) patients were on combination therapy with azathioprine or 6-mercaptopurine and a biologic

KTR= Renal transplant recipients. GD=Glomerular disease. IBD=Inflammatory bowel disease.

Table S5. Proportions of patients having been informed about their elevated skin cancer risk due to the immunosuppression. The publications are chronologically ordered by assessment date in the corresponding studies.

Publication	N	Informed about skin cancer risk
Cowen and Billingsley (1999)	122	41% of patients (n=50) did not recall learning specifically about an increased risk of skin cancer from these medications
Mahé et al. (2004)	445	91% of respondents remembered that they have been informed of the need for sun protection
Szepietowski et al. (2005)	151	113 (74.8%) patients were informed about the necessity of sun protection
Ismail et al. (2006)	292	96% (85% from dermatologist)
Clowers-Webb et al. (2006)	183	26% of the patients (n=48) indicated that they had not received information previously
Leung et al. (2018)	179	Post-transplantation, KTR received information about skin cancer either by verbal advice from a clinician (89%)[a] and/or by written information or pamphlets (74%), a lecture (29%), instructional videos (7%) or other means (7%)
Imko-Walczuk et al. (2016)	182	13 patients (7.1%) never received information about how to protect themselves against sunlight, 29 patients (15.9%) received the information once, 75 patients (41.2%) received the information several times, 32 patients (17.6%) received the information many times, and 33 patients (18.1%) did not remember ever receiving the information
Coughlin et al. (2017)	20	Have you discussed your child's risk for skin cancer with anyone (post-transplant)? yes: 10 (50%)
Walker et al. (2017)	149	133 respondents (90.5%)
Haney et al. (2019)	104	68 (65.4%) had not received training on the effect of sun exposure after the transplant, 28 (26.9%) had received training from a doctor
Patel et al. (2017)	40	27.5% of patients reported receiving educational information on skin cancer risk while being evaluated for their transplant
Tunçer Vural et al. (2018)	70	Yes: 39 (55.7%)<br>-by a dermatologist: 26 (37.1%)<br>-by a nurse: 13 (18.6%)
Sarigöl Ordin et al. (2023)	113	Yes: 54 (47.8%) ; 59 (52.2%) had not received information about the dangers of post-transplant sun exposure
Shope et al. (2023)	200	Yes: 122 (61%); No: 78 (39%)
Herlihy et al. (2024)	87	Yes: 34 (39%); No: 43 (49.5%); Unsure: 10 (11.5%)
Ali & Cronin (2025)	52 (HIV-positive: 27, HIV-negative: 25)	HIV-positive: Yes: 14 (52%) HIV-negative: Yes: 20 (80%)

KTR= Renal transplant recipients. [a] This value was used in the meta-analysis.

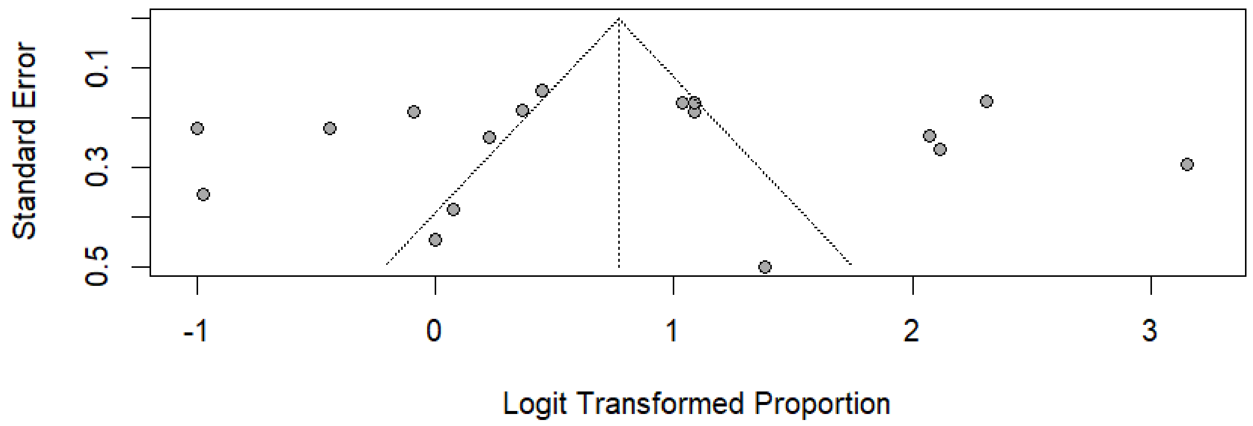

Figure S1: Funnel plot for the outcome "informed about skin cancer risk" (*n*=17*). *The study of Ali & Cronin (Ali & Cronin, 2025) is included with two samples.

Table S6. Skin cancer knowledge scores. The publications are chronologically ordered by assessment date in the corresponding studies.

Publication	N	Items	Results	Note
Clowers-Webb et al. (2006)	Number of patients who answered 75% of the items: 181 of 202	Patients were instructed to indicate whether each of the 18 statements was correct or incorrect. For each patient, the knowledge assessment was scored by calculating the percentage of correct responses among the answered items. Only patients who responded to at least 75% of the statements received a score.	mean (%) ± SD: 91.7 ± 8.3	In particular, most of the patients had 90% to 100% correct by having all of the statements correct or only 1 incorrect. Therefore, to provide a more meaningful description of the scores, the scores were categorized and summarized as 90% to 100%, 80% to 89%, 70% to 79%, and less than 70% correct. At baseline, 114 (63%) of the 181 patients had 90% to 100% correct answers
Coughlin et al. (2017)	pOTR: 20 Guardians: 20	Knowledge score (6/11 items skin cancer related)	pOTR: mean=4.67 Guardians: mean=6.58	
Haney et al. (2019)	104	SCSK (0-25)	mean ± SD: 10.98 ± 4.64	
Sorensen et al. (2018)	25	SCSK (0-25)	mean=16.5	
Sarigöl Ordin et al. (2023)	113	SCSK (0-25)	mean ± SD: 12.17 ± 2.84	The item for which OTR showed the highest level of knowledge was, "Skin cancer is not seen in people with dark skin". No participants gave the correct answer to 2 items on the questionnaire: "What types of clothing block UV rays (from the sun)?" and "What is the most common type of skin cancer?
Thet et al. (2022)	50	SCSK (0-25)	mean ± SD: 19.3 ± 3.3	KTR: mean=19.7 (+-3.1), GD: mean=19.0 (+-3.5)

SD=Standard deviation. pOTR=pediatric organ transplant recipient. SCSK=Skin Cancer and Sun Knowledge. OTR=Organ transplant recipients. KTR=Kidney transplant recipients. GD=Glomerular disease.

Table S7. Other items. The publications are chronologically ordered by assessment date.

<table>
<tr><th>Publication</th><th>N</th><th>Items</th><th colspan="2">Results (n [%])</th></tr>
<tr><td>Tavadia et al. (2006)</td><td>118</td><td>Skin cancer is a common form of cancer in the general population</td><td colspan="2">Agree: 79%; Disagree: 4%
Don't know: 17%</td></tr>
<tr><td rowspan="3">Kimmel et al. (2016)</td><td rowspan="3">164</td><td>Skin cancer can be healed without treatment</td><td colspan="2">False: 98.2%; True: 1.8%</td></tr>
<tr><td>Skin cancer can be cured if treated early</td><td colspan="2">True: 7.3%; False: 92.7%</td></tr>
<tr><td>Skin cancer can lead to death if not treated</td><td colspan="2">True: 1.2%; False: 98.8%</td></tr>
<tr><td rowspan="4">Wang et al. (2023)</td><td rowspan="4">POC: 118
Non-POC: 101</td><td rowspan="2">Skin cancer can appear on…</td><td>POC</td><td>Non-POC</td></tr>
<tr><td>Both: 93 (78.8%)
Areas exposed to the sun: 25 (21.2%)
Areas hidden from the sun: 0 (0%)</td><td>Both: 78 (77.2%)
Areas exposed to the sun: 23 (22.8%)
Areas hidden from the sun: 0 (0%)</td></tr>
<tr><td>There is not much I can do to lower my chances of getting skin cancer</td><td>Strongly agree: 12 (10.2%)
Somewhat agree: 10 (8.5%)
Neutral: 13 (11%)
Somewhat disagree: 28 (23.7%)
Strongly disagree: 55 (46.6%)</td><td>Strongly agree: 2 (2%)
Somewhat agree: 7 (6.9%)
Neutral: 6 (5.9%)
Somewhat disagree: 23 (22.8%)
Strongly disagree: 63 (62.4%)</td></tr>
<tr><td>I feel confident in recognizing suspicious skin growths or skin cancer</td><td>Strongly agree: 49 (41.5%)
Somewhat agree: 40 (33.9%)
Neutral: 9 (7.6%)
Somewhat disagree: 14 (11.9%)
Strongly disagree: 6 (5.1%)</td><td>Strongly agree: 38 (37.6%)
Somewhat agree: 44 (43.6%)
Neutral: 10 (9.9%)
Somewhat disagree: 3 (3%)
Strongly disagree: 6 (5.9%)</td></tr>
<tr><td>Farahbakhsh et al. (2024)</td><td>107</td><td>Knows what skin cancer looks like</td><td colspan="2">Abdominal transplant without skin cancer history: 39.6%
Abdominal transplant with positive skin cancer history: 66.7%
Thoracic transplant without skin cancer history: 50%
Thoracic transplant with positive skin cancer history: 85.7%</td></tr>
<tr><td>Herlihy et al. (2024)</td><td>82</td><td>How worried would you by a mole that… [sic!]</td><td colspan="2">
<table>
<tr><th>Characteristic</th><th>Not worried</th><th>A little worried</th><th>Quite worried</th><th>Very worried</th></tr>
<tr><td>Changing in colour</td><td>6 (7.3%)</td><td>33 (40.2%)</td><td>23 (28%)</td><td>20 (24.4%)</td></tr>
<tr><td>Increasing in size</td><td>6 (7.3%)</td><td>22 (27.2%)</td><td>30 (37%)</td><td>23 (28.4%)</td></tr>
<tr><td>With more than one colour</td><td>6 (7.3%)</td><td>21 (25.6%)</td><td>32 (39%)</td><td>23 (28%)</td></tr>
<tr><td>With irregular borders</td><td>6 (7.3%)</td><td>28 (35.1%)</td><td>25 (31.3%)</td><td>21 (26.3%)</td></tr>
<tr><td>Bigger than 6 mm</td><td>6 (7.3%)</td><td>22 (27.3%)</td><td>26 (32.1%)</td><td>27 (33.3%)</td></tr>
<tr><td>Is painful</td><td>4 (4.9%)</td><td>11 (13.6%)</td><td>31 (38.3%)</td><td>35 (43.2%)</td></tr>
<tr><td>Is itchy</td><td>6 (7.3%)</td><td>14 (16.9%)</td><td>32 (38.6%)</td><td>31 (37.3%)</td></tr>
<tr><td>Bleeding</td><td>6 (7.3%)</td><td>10 (12%)</td><td>27 (32.5%)</td><td>40 (48.2%)</td></tr>
</table>
</td></tr>
</table>

Parvathala et al. (2024)	40	I know what to look for when checking myself for skin cancer.	Strongly agree: 5 (12.5%), Agree: 18 (45.0%), Uncertain: 14 (35.0%), Disagree: 1 (2.5%), Strongly disagree: 2 (5.0%)

POC=People of color.
